# Supplementary material for: Learning curve and surgical outcome of robotic assisted colorectal surgery with ERAS program
Source: Sci Rep. 2022 Nov 29;12:20566. doi: 10.1038/s41598-022-24665-w (PMC9709162; doi:10.1038/s41598-022-24665-w)
Supplement: Supplementary file 1 — Supplementary Information 1. [file 41598_2022_24665_MOESM1_ESM.zip › LSC Raw data-Ñ[▒K/146/P_ñw╝╨░O▒Kñσ.pdf]

[redacted]

Pathologic diagnosis:
1. Sigmoid colon, laparoscopic anterior resection ---
Diverticulitis.
2. IMA root, dissection --- Unremarkable fibroadipose tissue, vessel
and lymph node.
3. Colon, cut end, distal, resection --- Hyperplastic polyp.

Gross description:
The specimen consists of 1) a segment of colon, 16 cm in length and 5.5cm and 4.5cm in bilateral cut end circumference, and mesocolic soft tissue. There is no obvious tumor or polyp noted grossly. On cross section, several diverticula are noted with associated inflammation with serosa involvement, with the distance of 4.5cm to the nearest cut end. The both cut ends are free of tumor grossly. 2) IMA root, 4 pieces of tan yellow soft tissue, 1.5x0.6x0.5 cm. 3) distal cut end, a piece of tan mucosa-covered soft tissue, 2x1.4x0.8 cm. Representative part for section: A-B) bilateral cut end C) diverticulum D) diverticulum with serosa involvement E-G) diverticula
All for section: H) specimen 2 J) specimen 3.
#T-59300_2 #M-40630_2 0 1105 000000
#T-59600_2 #M-72042_2 0 1105 000000

已電子簽章

敬告：本單僅供醫師參考使用，不具任何申請效力！！
請病患妥為保管，若不慎遺失需自負個人資料外洩責任！
